# Novel Garlic Carbon Dot-Incorporated Starch Whey Protein Emulsion Gel for Apple Spoilage Sensing

**DOI:** 10.3390/gels12010047

**Published:** 2026-01-01

**Authors:** Hebat-Allah S. Tohamy

**Affiliations:** Cellulose and Paper Department, National Research Centre, 33 El Bohouth Str., Dokki, Giza P.O. Box 12622, Egypt; hebasarhan89@yahoo.com

**Keywords:** electrostatic mapping, DFT calculations, red garlic peels, colored carbon dots, whey protein, food spoilage, intelligent packaging, biosensor

## Abstract

This study presents the development of a smart packaging material utilizing garlic-derived nitrogen-doped carbon dots (CDs) integrated into a whey protein–starch (WP-S) emulsion. The research aimed to create a real-time, non-invasive biosensor capable of detecting microbial spoilage. The synthesized CDs demonstrated strong pH-sensitive photoluminescence, exhibiting distinct changes in CIE coordinates and fluorescence intensity in response to varying pH values. The WP-S-CDs emulsion was tested against *E. coli*, *S. aureus*, and *C. albicans*. The results showed that the composite film provided a clear colorimetric shift and fluorescence quenching, both of which are directly correlated with microbial metabolic activity. The physical and electronic properties of the composite were investigated to understand the sensing mechanism. Scanning electron microscopy (SEM) of the dried film revealed that the WP-S-CDs system formed a more porous structure with larger pore sizes (3.63–8.18 µm) compared to the control WP-S film (1.62–6.52 µm), which facilitated the rapid diffusion of microbial metabolites. Additionally, density functional theory (DFT) calculations demonstrated that the incorporation of CDs significantly enhanced the composite’s electronic properties by reducing its band gap and increasing its dipole moment, thereby heightening its reactivity and sensitivity to spoilage byproducts. In a practical application on apples, the WP-S-CDs coating produced a visible red spot, confirming its function as a dynamic sensor. The material also showed a dual-action antimicrobial effect, synergistically inhibiting *C. albicans* while exhibiting an antagonistic effect against bacteria. These findings validate the potential of the WP-S-CDs emulsion as a powerful, multi-faceted intelligent packaging system for food quality monitoring.

## 1. Introduction

The global food supply chain faces a persistent challenge in ensuring the quality and safety of perishable products, as microbial contamination and subsequent spoilage lead to significant economic losses and potential health risks [1,2,3,4,5]. Traditional methods for detecting spoilage, such as microbial culture counting and chemical analysis, are often slow, costly, and destructive, requiring samples to be taken to a laboratory for analysis [6,7,8,9]. This time-consuming process hinders real-time monitoring and prevents swift intervention to mitigate spoilage, thereby creating a critical need for new, effective, and non-invasive detection technologies [10,11,12,13,14]. In recent years, intelligent food packaging systems have emerged as a promising solution, offering the ability to provide real-time information about food quality [15,16,17,18,19]. These systems are designed to interact directly with the food or its headspace and provide a visual or electronic signal indicating the state of freshness [16,20]. However, many existing intelligent packaging materials rely on synthetic indicators or lack the multifunctional properties necessary for a truly comprehensive system [21,22,23,24,25].

Garlic peel waste (GP), often discarded as an inedible byproduct of garlic processing, presents a significant environmental and economic challenge [26]. With millions of tons of this waste generated annually, its conventional disposal in landfills or through burning contributes to soil and air pollution, while also representing a massive loss of valuable resources [27,28,29,30,31]. The high organic content of garlic peels in landfills can generate greenhouse gases, and open-air burning releases harmful particulate matter into the atmosphere [20,32]. The importance of recycling this waste lies in its rich composition. Far from being a mere byproduct, garlic peels are a reservoir of valuable bioactive compounds. They contain significant amounts of polyphenols, flavonoids, and organosulfur compounds, which possess powerful antioxidant, antimicrobial, and anti-inflammatory properties [5,33]. By upcycling this waste, we can extract these beneficial components for use in various industries. For example, the peels can be repurposed as a natural source for carbon dots (CDs), which can then be used in advanced sensors, or as a natural pesticide and fertilizer for sustainable farming. This approach aligns with the principles of a circular economy, transforming a discarded material into a new, high-value product and mitigating the environmental impact of agricultural waste. Building on their unique properties, CDs have emerged as a versatile class of nanomaterials with a wide range of applications. Their small size, typically less than 10 nanometers, and surface functionalization capabilities allow them to be used in various fields [34,35,36,37,38]. For instance, in bioimaging, CDs can be conjugated with targeting molecules to selectively label and visualize specific cells or tissues. Their tunable fluorescence also makes them excellent candidates for advanced diagnostic tools and biosensors. Furthermore, the high biocompatibility of CDs means they can be safely introduced into biological systems, which is a major advantage over traditional heavy-metal-based quantum dots [39,40,41]. These attributes make CDs ideal candidates for various sensing applications, including those in the food industry. The unique properties of both the biopolymer matrix and the CDs present an opportunity for synergy.

In the pursuit of sustainable and effective food packaging, natural biopolymers such as whey protein (WP) and starch (S) have garnered significant attention due to their biodegradability, excellent film-forming properties, and ability to act as natural barriers [42,43,44,45,46,47,48,49,50,51]. Starch is a polymeric carbohydrate composed of D-glucose units linked primarily by α-1,4 glycosidic bonds [52,53,54]. This polymer can be further divided into two main components: amylose and amylopectin, which together constitute 98–99% of the dry mass of starch [55,56,57]. Amylose is a linear polysaccharide chain of D-glucose units. In contrast, amylopectin is a highly branched polysaccharide with a backbone of α-1,4 glycosidic bonds and branches formed by α-1,6 glycosidic bonds. These two different structures pack together to form semi-crystalline and amorphous layers arranged in concentric rings, which gives the starch granule its characteristic structure [44,54,58]. Whey is a dairy industry by-product derived from cheesemaking and casein manufacturing, and it makes up about 20% of the total protein in milk. Due to its high concentration of lactose (over 75% of total whey solids), dairy whey is considered one of the most polluting food by-products. This is because it has a very high biochemical oxygen demand and a chemical oxygen demand, both of which indicate its significant environmental impact [57,59]. After its collection and processing, whey protein (WP) can be isolated from the whey. WP is a highly valuable nutritional ingredient because of its excellent amino acid profile and high bioavailability. It is a complete protein, meaning it contains all nine essential amino acids necessary for human health [60,61,62]. As abundant and low-cost materials, they represent a compelling alternative to conventional petroleum-based plastics. However, these often lack the advanced functionalities required for real-time food quality monitoring. Concurrently, the emerging field of CDs has introduced a new class of nanomaterials with exceptional optical properties, high biocompatibility, and tunable fluorescence [63,64]. In the field of edible and biodegradable packaging, the application method of a material is as critical as its composition. While pre-formed films provide a convenient barrier, they often face limitations in providing intimate contact with food products, especially those with irregular shapes, leading to compromised barrier integrity and inconsistent surface coverage. This challenge can be effectively overcome by using a film-forming solution in the form of an emulsion [65,66,67,68]. The liquid nature of an emulsion allows for a direct-coating approach, enabling the material to be applied directly to the food surface as a thin, continuous layer that conforms to its contours. This not only creates a superior and more uniform barrier against moisture and oxygen but also allows for a more direct and efficient interaction between the film’s active components and the food’s surface [69,70,71,72]. Consequently, the use of an emulsion is paramount for developing intelligent packaging systems that require close physical and chemical contact to sense and report on the food’s condition in real time. In our study, by combining WP and S, it is possible to create a composite system that leverages the robust structural qualities of the WP-S emulsion while integrating the dynamic, sensitive properties of the CDs. The resulting material is a multi-functional bio-nanocomposite with enhanced electronic and physical characteristics. This study investigates the synthesis and characterization of a novel WP-S film integrated with nitrogen-doped carbon dots (WP-S-CDs). This study investigates the synthesis and characterization of a novel whey protein–starch emulsion integrated with nitrogen-doped carbon dots (WP-S-CDs) derived from garlic waste. The primary research aim is to develop and validate a real-time, multi-faceted intelligent packaging biosensor capable of non-invasively detecting microbial spoilage on fresh produce (apples) by utilizing the pH-responsive chromogenic and fluorescent properties of the N-CDs, while simultaneously evaluating the system’s dual-action antimicrobial performance.

## 2. Results and Discussion

### 2.1. Chromaticity and Optical Properties of Garlic-Derived Carbon Dots at Different pH Values and WP-S-CDs Emulsions Before and After Contact with Different Types of Bacteria for Differentiation

**Figure 1:**
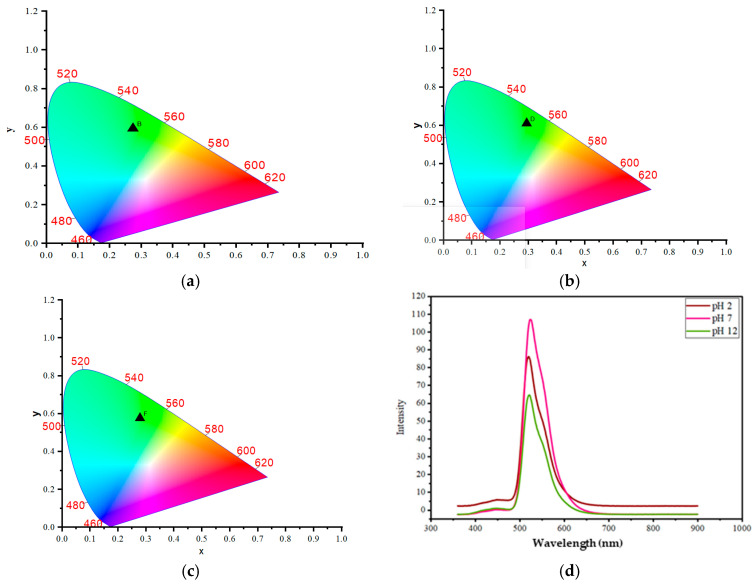
CIE of the CDs in the chromaticity diagram (**a**) at pH 2, (**b**) pH 7, (**c**) pH 12; and (**d**) The fluorescent spectroscopy study of CDs.

To establish the fundamental sensing mechanism, we first investigated the intrinsic pH-sensitivity of the synthesized carbon dots (CDs) in solution. The CIE coordinates of the resultant emission confirmed the CDs’ sensitivity to pH (Figure 1a–c). The coordinates, which define the perceived color of the emission, show a clear shift from acidic to neutral to alkaline conditions. The initial emission at pH 2 is a bluish-green, represented by coordinates (0.27, 0.59). This suggests a strong green component with a slight blue tint. As the pH becomes neutral at pH 7, the coordinates shift to (0.29, 0.61). This represents a significant move towards a purer green, as the increase in both the x and y coordinates places the color closer to the central green region of the CIE diagram. This change is often attributed to the protonation of surface functional groups on the CDs, such as carboxyl or hydroxyl groups. The acidic environment causes these surface groups to become protonated, which changes the electron density and energy levels on the CD’s surface, influencing the emission color. At pH 12, the coordinates move back to (0.27, 0.57). This is very close to the emission at pH 2, and suggests the emission color is once again a bluish-green, similar to the acidic condition but with a slightly weaker green character. The highly alkaline environment causes these same surface groups to become fully deprotonated. This deprotonation state, while chemically opposite to the protonation at pH 2, can lead to a similar electronic configuration on the CD surface [15].

To precisely attribute the source of the composite’s luminescent activity, we performed a comparative spectrofluorimetry analysis between the s CDs, the pure Whey Protein–Starch (WP-S) matrix, and the final WP-S-CDs bio-emulsion. The pure WP-S film exhibited negligible native fluorescence across the excitation and emission ranges relevant to the CDs, confirming that the biopolymer matrix itself is optically transparent and non-emissive under these conditions. This crucial comparison establishes that the strong dual-peak photoluminescence observed in the WP-S-CDs composite with the primary emission peak near 440 nm and the secondary peak near 500 nm is derived entirely from the integrated garlic-derived CDs. The WP-S matrix serves solely as a structural host, embedding the CDs without significant internal quenching or spectral interference. This finding validates our subsequent analysis that all observed pH-sensitive colorimetric shifts and fluorescence quenching effects in the WP-S-CDs biosensor are direct, traceable responses mediated by the surface functional groups of the nitrogen-doped CDs interacting with the microbial metabolites. The fluorescent spectroscopy study was then conducted to provide a quantitative analysis of the CDs’ optical properties and their response to pH changes. The synthesized CDs demonstrated distinct optical properties and pH sensitivity, as shown in the provided data. When excited at a wavelength of 350 nm, the CDs exhibited two distinct emission peaks, confirming the presence of multiple luminescent centers. The primary emission peak was consistently observed in the blue-violet region, appearing at 445 nm (pH 2), 444 nm (pH 7), and 443 nm (pH 12). This emission is primarily attributed to the inherent luminescence from the C=O and C=N surface moieties present due to the nitrogen and oxygen doping [5,73]. In addition to this main peak, a second, longer-wavelength emission peak was also present, consistently observed in the green region at 521 nm (pH 2), 524 nm (pH 7), and 522 nm (pH 12) (Figure 1d). This shifted emission is ascribed to oxygen vacancy defects within the CDs structure. A crucial finding was the significant change in peak intensity with varying pH values. The overall fluorescence intensity increased from pH 2 to pH 7 and then began to decrease at pH 12. This behavior is indicative of the CDs’ sensitivity to changes in the surrounding proton concentration. The intensity enhancement at neutral pH can be attributed to the deprotonation of surface functional groups, which reduces their ability to quench fluorescence. Conversely, the quenching effect observed at high (alkaline) pH value suggests that the interaction with excess hydroxide ions can alter the PL properties. This responsiveness to pH change validates the CDs’ potential for use as a pH-sensitive sensor.

Based on the precursor material (red garlic peels, which are rich in carbon, nitrogen, and sulfur compounds) and the comprehensive characterization data, we propose a representative structural model for the synthesized CDs. The core of the CD is presumed to be a small, graphitic-like sp2 carbon lattice, which is responsible for the primary π-π transition giving rise to the shorter-wavelength photoluminescence (PL) peak (near 440 nm). However, the CD surface is heavily functionalized, resulting in nitrogen-doped CDs (N-CDs). This surface functionality is key to the material’s properties, introducing oxygen groups (carboxyl and hydroxyl) for high hydrophilicity and, more importantly, nitrogen groups (such as amine and pyridinic/pyrrolic species). These N-containing groups, particularly the C=N and C–N bonds, serve as the primary pH-sensing sites. The exceptional pH-sensitivity is directly attributed to the protonation and deprotonation of these groups, which modulates the electron density and surface energy states. This modulation facilitates the n-π* transition that gives rise to the secondary, longer-wavelength PL peak (near 500 nm) and is the fundamental mechanism driving the fluorescence quenching and colorimetric shift observed upon interaction with the alkaline microbial metabolites, thereby validating the high sensitivity of the WP-S-CDs biosensor.

**Figure 2:**
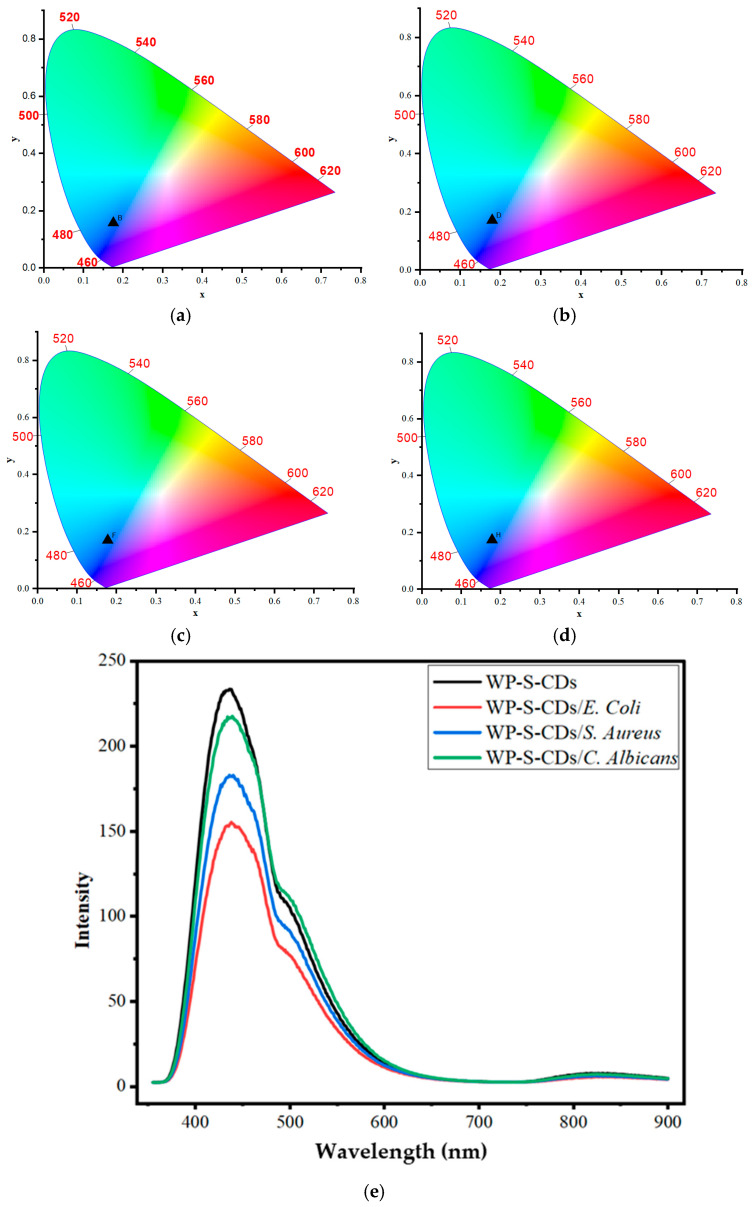
CIE of the CDs in the chromaticity diagram for (**a**) WP-S-CDs, (**b**) WP-S-CDs/*E. coli*, (**c**) WP-S-CDs/*S. aureus*, (**d**) WP-S-CDs/*C. albicans*; and (**e**) The fluorescent spectroscopy study of WP-S-CDs, WP-S-CDs/*E. coli*, WP-S-CDs/*S. aureus*, and WP-S-CDs/*C. albicans*.

Having confirmed the pH-sensitivity of the standalone CDs, we proceeded to test the performance of the integrated WP-S-CDs bio-emulsion against common food spoilage microorganisms. The prepared WP-S-CDs bio-emulsion showed a distinct CIE color change in response to different microbial contaminants, indicating its potential as a colorimetric biosensor for spoilage (Figure 2a–d). The initial CIE coordinates for the WP-S-CDs emulsion were (0.175, 0.158). This is a noticeable shift from the coordinates of the standalone carbon dots (CDs) which we previously discussed above, which were in the bluish-green region. This change in baseline color is likely due to the interaction between the nitrogen-doped CDs and WP-S emulsion. The surrounding polymer environment can affect the electron states and surface functional groups of the CDs, causing a shift in their fluorescence emission. When the WP-S-CDs emulsion came into contact with the different microorganisms, a distinct change in the CIE coordinates occurred. For WP-S-CDs/*E. coli*, the coordinates shifted to (0.178, 0.171). For WP-S-CDs/*S. aureus*, the coordinates shifted to (0.177, 0.170). For WP-S-CDs/*C. albicans*, the coordinates shifted to (0.177, 0.174). All three contaminated samples showed a shift from the original coordinates toward higher x and y values. This indicates a move from the initial blue color towards a bluish-green or even cyan hue on the CIE chromaticity diagram. This color change is a direct result of the interaction between the CDs and the metabolic byproducts of the microorganisms. Bacteria and fungi like *E. coli*, *S. aureus*, and *C. albicans* produce various metabolites, including organic acids, bases, or other substances that alter the local chemical environment (particularly the pH) surrounding the nitrogen doped CDs (N-CDs). As we previously noted in the CIE graphs, the CDs are highly sensitive to pH changes due to the protonation and deprotonation of their surface functional groups (like C=O and C=N). The fact that the coordinate shifts are similar for all three microorganisms suggests a common sensing mechanism, most likely a change in the local pH. The slight differences in the degree of shift could be attributed to variations in the specific metabolic byproducts and their concentrations produced by each microorganism. This confirms the potential of the WP-S-CDs bio-nanocomposite as a powerful tool for intelligent packaging, as it can visually signal the presence of microbial spoilage.

We have also assessed the fluorescence intensity and peak wavelength shifts to provide a quantitative measure of the microbial contamination, and the color. Beyond the color shift, the fluorescence intensity data provides a quantitative measure of the microbial contamination. The overall fluorescence intensity from PL of the WP-S-CDs system decreased in the presence of all three microorganisms (Figure 2e). The order of decreasing intensity was WP-S-CDs > WP-S-CDs/*C. albicans* > WP-S-CDs/*S. aureus* > WP-S-CDs/*E. coli*. This finding is particularly significant because it confirms that the microbes are producing substances that actively quench the fluorescence of the CDs. Based on the standalone pH study, this quenching is likely caused by the production of alkaline byproducts, which lead to the deprotonation of the N-CDs’ surface functional groups. The degree of quenching is directly related to the amount of metabolic activity, allowing for the semi-quantitative detection of contamination. The degree of quenching correlates directly with the magnitude of this effect, suggesting that *E. coli* produces the most significant amount of fluorescence-quenching byproducts, followed by *S. aureus*, and then *C. albicans*. This quantitative difference in quenching not only confirms that spoilage is occurring but also provides an indication of its severity. The initial WP-S-CDs emulsion has two main emission peaks: a primary peak at 439 nm and a secondary peak at 501 nm. After contact with the microbes, a subtle but distinct shift in these peak positions occurs. For *E. coli*, the first peak shows a notable red shift to 444 nm, while for *S. aureus*, it shifts to 441 nm. The *C. albicans* sample shows virtually no shift in the first peak, but the second peak slightly red-shifts to 502 nm. These varying shifts in peak position, known as bathochromic shifts (red shifts), confirm that each microbe’s metabolic byproducts are altering the chemical environment around the CDs in a slightly different way. This is a powerful finding because it suggests the sensor is not just detecting a general change, but is potentially sensitive to the specific type of organism present, based on its unique metabolic fingerprint. In conclusion, the developed WP-S-CDs bio-emulsion serves as a highly effective, multi-faceted sensor. It not only provides a visual, colorimetric signal of microbial contamination but also offers a quantitative indication of its severity through fluorescence quenching. This demonstrates the immense potential of this intelligent packaging system to provide real-time, non-invasive monitoring of food quality.

### 2.2. Apple Spoilage Sensing by WP-S-CDs with Antimicrobial Properties

**Figure 3:**
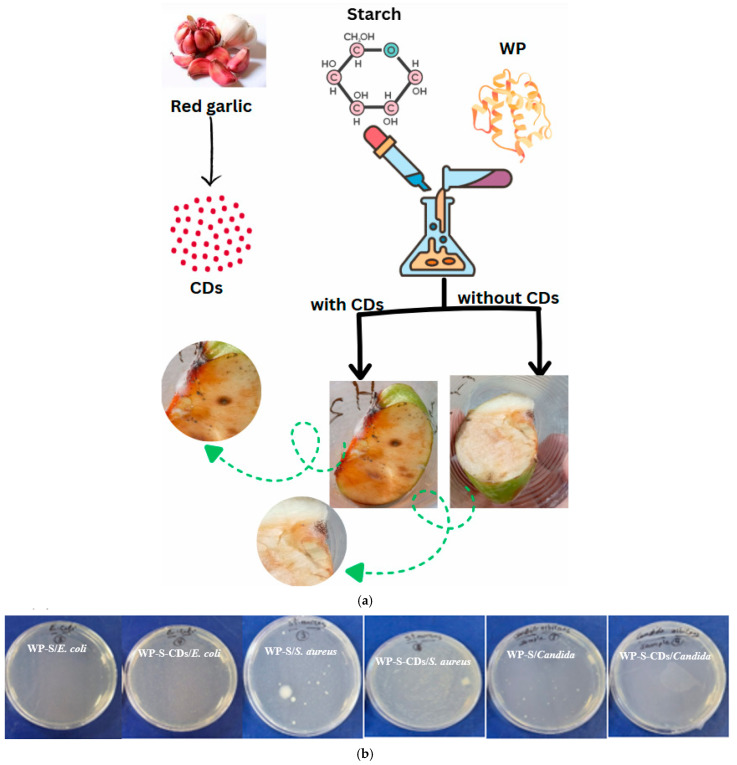
(**a**) Application of WP-S-CDs emulsion in apple’s preservation and spoilage detection by naked eye response; and (**b**) antimicrobial activity of WP-S (denoted as 3) and WP-S-CDs (denoted as 4) against different types of bacteria and fungi.

Following the successful in vitro testing against specific microorganisms, we conducted a practical evaluation of the WP-S-CDs coating’s ability to sense spoilage on a real food product, the apple. The observation of a red spot on the WP-S-CDs-coated apple, directly associated with bacterial colonies, provides a powerful and visually clear validation of the sensor’s functionality (Figure 3a). In contrast, the WP-S made just a bacterial colony without any sensing observation. The key to understanding this phenomenon lies in the fact that this CDs are nitrogen-doped. The incorporation of nitrogen creates specific functional groups, such as C=N bonds, on the surface of the carbon dots. As previously discussed, these very groups are responsible for the dual-peak PL we observed above and the remarkable pH-sensitivity of the CDs. The appearance of a red spot represents a more extreme and significant event along the PL peaks shift which discussed above. It is a severe red shift in the PL emission, likely caused by a combination of factors. The robust microbial activity on the apple’s surface has likely produced highly alkaline byproducts, causing a pronounced deprotonation of the nitrogen doped CDs’ surface groups. This change in surface chemistry is so dramatic that it shifts the PL emission to a much longer wavelength, resulting in the visible red color. In essence, the red spot is a strong visual signal that the CDs have reached a new state of deprotonation in response to significant microbial contamination, confirming their high sensitivity and potential as a dynamic biosensor in a complex food system.

Next, we evaluated the dual-action antimicrobial properties of the WP-S and WP-S-CDs coatings against the three tested microbial strains. The CFU data shows that the control coating, WP-S, possesses a strong inherent antimicrobial effect on its own. It achieved significant inhibition against all three microbial strains, with a remarkable 95.71% effectiveness against *S. aureus* and 81.42% against *E. coli*. This is likely due to the intrinsic properties of the WP itself, which contains known antimicrobial peptides like lactoferrin and lactoperoxidase [71,72]. The physical barrier created by the WP-S matrix may also contribute by limiting oxygen availability or dehydrating the microbial cells, further impeding their growth (Figure 3b; Table 1). The addition of the N-CDs created a selective and somewhat counterintuitive antimicrobial effect. Instead of a uniform increase in efficacy, the CDs exhibited a dual-action response. For the bacteria, a decrease in antimicrobial activity was observed. The effectiveness against *E. coli* dropped from 81.42% (WP-S) to 67.44% (WP-S-CDs), and against *S. aureus*, it plunged drastically from 95.71% to just 49.42%. This is a surprising finding, as active nanoparticles are typically expected to enhance antimicrobial properties. A plausible explanation is that the nitrogen doped CDs are interacting with the antimicrobial peptides in the whey protein, binding to them or altering their structure and thereby reducing their effectiveness against these specific bacterial strains. This suggests an antagonistic effect between the CDs and the antimicrobial components of the WP-S matrix. However, the addition of CDs resulted in a dramatic improvement in efficacy against the yeast *C. albicans*, with the inhibition rate soaring from 68.54% (WP-S) to an impressive 96.63% (WP-S-CDs). This finding strongly suggests a synergistic effect between the CDs and the WP-S matrix. CDs are known to disrupt fungal cell membranes and interfere with their metabolic pathways, and it appears the nitrogen doped CDs have a particularly potent antifungal effect that is significantly enhanced by their incorporation into the bio-nanocomposite.

The observed antagonistic effect against bacteria is acknowledged as a critical finding and a subject for future optimization. It is important to emphasize that the primary objective of the hybrid WP-S-CDs is to function as a highly sensitive, real-time biosensor for spoilage detection, a goal which was successfully demonstrated (Section 2.1 and Section 2.2). The trade-off in bacterial inhibition is a direct consequence of integrating the N-CDs in a way that prioritizes sensor functionality and high sensitivity. The electronic changes (reduced band gap, enhanced dipole moment) that make the film a highly effective sensor concurrently appear to interfere with the antibacterial mechanism of the WP. However, the resulting system still presents a powerful dual-action profile through its high sensitivity to spoilage byproducts and its dramatic synergistic antifungal capability. Our future work to restore antibacterial efficacy and to address this selectivity and restore full-spectrum antimicrobial activity, future research will focus on optimizing the formulation to prevent the antagonism between the N-CDs and the WP peptides. Potential strategies include: (1) Encapsulation: Using nanocarriers to encapsulate the N-CDs or the WP peptides to physically separate the antagonistic components before blending. (2) Alternative Bactericidal Additives: Introducing a low concentration of a separate, non-interacting bacteriostatic agent (e.g., alternative natural extracts) to specifically enhance the antibacterial capacity without compromising the N-CDs’ sensing function. This work focuses on the novelty of the sensing mechanism; the next phase will ensure a comprehensive, non-antagonistic dual-action (sensing + full-spectrum antimicrobial) system.

### 2.3. DFT Calculations with the Electrostatic Mapping

**Figure 4:**
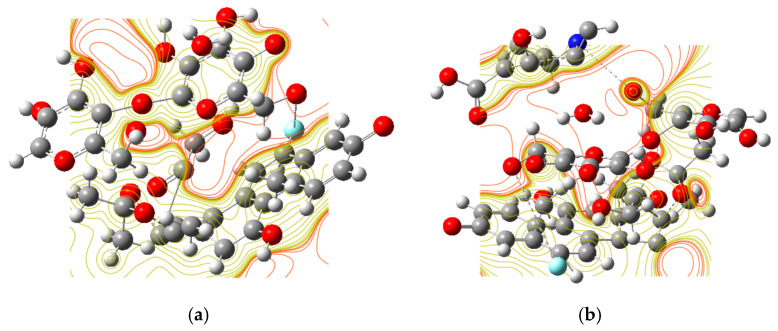
The ESPM for the (**a**) WP-S and (**b**) WP-S-CDs (red regions indicate negative potentials) and (nucleophilic-donor coordination sites).

The results from DFT calculations provide a powerful electronic and molecular-level explanation for the observed behavior of the WPS-S and WP-S-CDs emulsions (Figure 4, Table 2). The most striking finding is the significant decrease in the E_g_ from 0.1867 eV for the WP-S emulsion to 0.1633 eV for the WP-S-CDs emulsion. A smaller band gap signifies a lower energy requirement for electron excitation, which directly correlates with the WP-S-CDs’ enhanced reactivity. This heightened electronic activity is the fundamental reason for the WP-S-CDs’ ability to act as a sensitive biosensor [74]. The addition of the CDs makes the material more susceptible to electron transfer, allowing it to readily interact with the metabolic byproducts of microorganisms, which in turn leads to the observed colorimetric and fluorescence changes. The addition of the carbon dots resulted in a substantial increase in the μ, from 20.04 Debye for WP-S to 32.66 Debye for WP-S-CDs. This increase indicates a significant redistribution of charge within the WP-S-CDs, making it more polar. The enhanced polarity and charge separation facilitate stronger interactions with external chemical species. This is particularly relevant to the sensing mechanism, as microbial metabolites, which can be charged or polar, are more easily detected by a material with a higher dipole moment. The concept of σ also strongly supports this conclusion. The value for WP-S-CDs (−10.11 eV) is dramatically larger than that for WP-S (−0.1226 eV), confirming that the WP-S-CDs is a much “softer” material. Chemical softness is a measure of a WP-S-CDs’ ability to undergo charge transfer, and the drastic increase in this value explains the high sensitivity and dynamic response of the sensor to microbial contamination [15,73]. Finally, the DFT calculations confirm the thermodynamic stability of the composite system. The ET of the WP-S-CDs (−3313.06 a.u.) is significantly lower and thus more stable than that of the WP-S sample (−2733.35 a.u.). This finding is crucial because it demonstrates that the CDs are not simply suspended within the emulsion but are well-integrated into the polymer matrix, forming a stable bio-nanocomposite. This stability ensures that the sensor maintains its structural integrity and sensing functionality over time. In conclusion, the DFT analysis provides a robust theoretical foundation for the experimental results, showing that the nitrogen-doped CDs enhance the electronic properties, reactivity, and stability of the WP-S emulsion, thereby transforming it into a highly effective and dynamic intelligent packaging sensor.

The electrostatic potential maps (ESPM) provide a crucial visual and quantitative representation of the charge distribution on the surface of the films. The finding that the pure WP-S exhibits more pronounced “red lines”—indicating a higher concentration of localized negative charges—compared to the WP-S-CDs is highly significant, providing direct evidence of the fundamental electronic changes induced by the incorporation of the nitrogen-doped CDs into the WP-S emulsion. The native structure of the WP-S, with its constituent WP and S molecules, contains numerous deprotonated functional groups, such as carboxyl (–COO–), hydroxyl (–O–) groups, which are the source of these localized negative charges. The ESPM results show that in the pure WP-S, these charges are relatively concentrated and static. The introduction of the CDs fundamentally alters this electronic landscape. As a result of their nitrogen-doping, these CDs are known to have a strong electron-accepting capability and high chemical softness, as confirmed by DFT calculations. The ESPM data strongly suggest that the CDs act as charge-distributing agents within the WP-S matrix. Instead of remaining in localized areas, the negative charge from the protein and starch functional groups is delocalized and distributed across the surface of the carbon dots through electrostatic interactions and possible charge-transfer pathways. This process effectively “smears out” the concentrated negative regions, leading to a decrease in the intensity and number of the “red lines” observed in the ESPM of the WP-S-CDs composite. This electronic redistribution is the key to the composite’s enhanced functionality. By drawing in and stabilizing the negative charge, the CDs create a more reactive surface that is primed for interaction with external molecules. This is the very mechanism that facilitates the rapid and sensitive detection of volatile byproducts from microbial metabolism, which are responsible for the observed colorimetric shifts and fluorescence quenching. Therefore, the ESPM data provide direct, visual support for the theoretical predictions and experimental observations of the WP-S-CDs’ enhanced sensing properties.

### 2.4. TEM Analysis for CDs, SEM and Fourier Transform Infrared Spectroscopy (FTIR) Spectra

**Figure 5:**
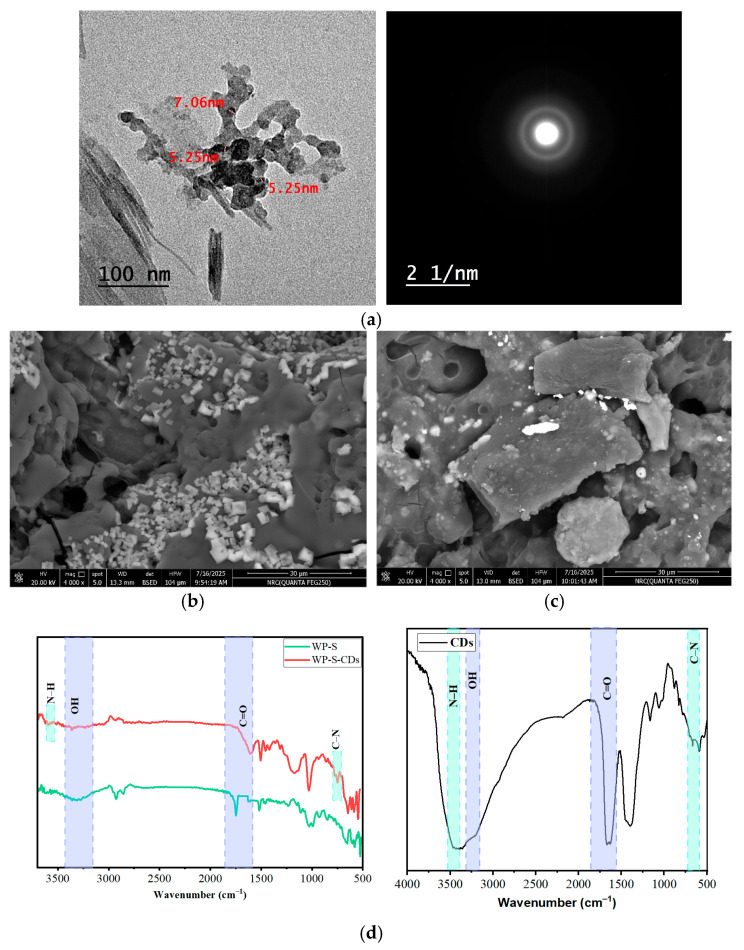
(**a**) TEM analysis with SAED for CDs, SEM analysis for (**b**) WP-S, (**c**) WP-S-CDsfor WP-S and WP-S-CDs; and (**d**) FTIR of CDs, WP-S and WP-S-CDs.

The TEM images of the purified CDs clearly reveal that the dots possess a distinct, near-spherical morphology. Crucially, the size distribution is narrow, with the measured average diameter of the synthesized CDs ranging specifically from 5.25 to 7.05 nm. This size range firmly establishes the material as nanoscale. Larger and irregular structures are observable. These structures are identified as aggregates or agglomerates and are common artifacts resulting from the rapid solvent evaporation during the preparation of the TEM grid. This drying process concentrates the highly dispersed particles, leading to localized clustering on the substrate. Importantly, the dimensions reported (5.25 nm and 7.06 nm as indicated) correspond to the diameters of the elementary, constituent primary CD particles observed at the periphery and within the aggregate’s structure, not the size of the aggregate itself. Furthermore, High-Resolution TEM (HR-TEM) analysis and Selected Area Electron Diffraction (SAED) confirm the crystalline nature of the CDs. The SAED pattern, characterized by concentric diffraction rings rather than sharp spots, indicates that the CDs are polycrystalline, consistent with randomly oriented nanometer-sized crystallites. This pattern validates the successful conversion of the GP precursor into the active carbon core structure (Figure 5a). The SEM results showing the difference in pore size between the two films after drying these two emulsions (WP-S and WP-S-CDs) directly explains the sensing mechanism for the apple spoilage (Figure 5b,c). The WP-S-CDs film, with its larger pore size (3.63–8.18 µm), creates a more porous and less dense structure compared to the WP-S film’s tighter, more compact structure (1.62–6.52 µm pore size). This higher porosity is not a drawback; rather, it is a deliberate and beneficial outcome of incorporating the CDs. The larger pores act as micro-channels, providing a direct and efficient pathway for volatile organic compounds (VOCs) produced by microbial metabolism to migrate from the apple’s surface into the film. These VOCs, which are the byproducts of spoilage, can then quickly reach the embedded nitrogen-doped CDs. The more direct access to the CDs allows for a faster and more pronounced reaction, leading to the rapid colorimetric and fluorescence changes which we have observed. In contrast, the WP-S control film, with its smaller pores, forms a denser barrier. This structure restricts the diffusion of microbial metabolites, limiting their interaction with any potential sensing elements. This is why the WP-S film only exhibited an antimicrobial effect (by physically impeding growth) but no sensing capability. In essence, the specific pore structure induced by the addition of the CDs transforms the film from a simple antimicrobial coating into a truly intelligent packaging system, enabling the real-time, visual detection of spoilage. The pore size is the physical key that unlocks the chemical sensing properties of the CDs. The FTIR proved the incorporation of CDs from the additional peaks which are appeared at 3590 and 744 cm^–1^ which are related to N–H and C–N, respectively, from the nitrogen doped CDs in WP-S-CDs (Figure 5d) [15,73].

## 3. Conclusions

Based on the comprehensive findings, this study successfully demonstrates the development of a highly effective, multi-functional whey protein–starch emulsion incorporating garlic-derived nitrogen-doped carbon dots (WP-S-CDs) for intelligent food packaging. The foundation of this system is the remarkable pH sensitivity of the synthesized carbon dots, which exhibit distinct changes in CIE color coordinates, photoluminescence intensity, and emission peak wavelengths in response to varying pH. When integrated into the WP-S emulsion, these CDs translate their intrinsic properties into a dynamic biosensor. The composite film provides a clear, visual signal of microbial contamination, as evidenced by a distinct shift in CIE coordinates from an initial blue to a bluish-green or cyan hue upon contact with different microorganisms. Beyond this qualitative colorimetric response, the system also offers a quantitative measure of spoilage severity through fluorescence quenching, with the degree of quenching directly correlating with the metabolic activity of the microbial contaminants. The high-performance sensing capability of the WP-S-CDs composite is supported by both theoretical and structural evidence. DFT calculations confirmed that incorporating the CDs significantly improved the WP-S-CDs’ electronic properties, notably by reducing the band gap, thereby enhancing its reactivity and susceptibility to electron transfer. The significant increase in the dipole moment and chemical softness further validates the composite’s heightened ability to interact with and sense microbial byproducts. Complementing these theoretical insights, SEM analysis revealed that the WP-S-CDs film possessed a larger pore size compared to the control. This more porous structure creates efficient micro-channels that allow volatile compounds produced by microbial metabolism to rapidly diffuse into the film, ensuring swift and real-time sensor activation. Furthermore, the bio-nanocomposite demonstrated a unique and dual-action antimicrobial profile. While the WP-S matrix inherently possesses a broad-spectrum antimicrobial effect, the addition of the CDs created a selective response. A surprising antagonistic effect was observed against bacteria (*E. coli* and *S. aureus*), leading to a reduction in antimicrobial efficacy. In contrast, the CDs exhibited a dramatic synergistic effect with the WP-S matrix, significantly enhancing the inhibitory action against the yeast *C. albicans*. This dual antimicrobial and sensing functionality, coupled with the proven stability and enhanced reactivity of the composite, establishes the WP-S-CDs system as a promising and versatile solution for non-invasive, real-time monitoring of food quality and safety.

## 4. Materials and Methods

### 4.1. Materials

Apple was purchased from Granada (Islamic Spain). The red garlic peels (GP) used in this study were obtained as a waste byproduct from a local Egyptian kitchen.

### 4.2. Preparation of Carbon Dots from Red Garlic Peels

Since the red garlic peels (GP) were obtained as an agricultural waste byproduct, a meticulous pretreatment process was essential to ensure material quality, remove contaminants, and guarantee the reproducibility of the synthesized carbon dots (CDs). First, collection and initial cleaning: GP were collected immediately after processing. The raw material was first subjected to extensive washing with deionized (DI) water to remove any surface dust, adhering soil particles, and soluble residues. Second, drying and milling: The washed peels were then oven-dried at 60 °C to remove all residual moisture and achieve a constant weight. The dried peels were ground into a fine powder. This step ensures a uniform particle size distribution, which is crucial for consistent pyrolysis and chemical reaction kinetics during synthesis. The N-doped carbon dots (N-CDs) were synthesized using a microwave-assisted method, which allows for rapid, scalable production and superior control over the doping process due to the rapid heating rate. The synthesis proceeded as follows: a mixture of 4 g GP, 4 g NaOH, 4 g urea, and 100 mL water. This mixture was then sonicated, frozen, and irradiated with a microwave at 700 W for 10 min. The final product, a clear, photoluminescent solution of purified N-CDs, was stored for subsequent use. This final purification step ensures that only the stable, small-sized CDs remain, enhancing the reproducibility of the luminescent response.

### 4.3. Preparation of Whey Protein–Starch Emulsions

Two separate samples were created for this study: a whey protein isolate-starch bio-nanocomposite emulsion with garlic-derived nitrogen-doped carbon dots (CDs), labeled WP-S-CDs, and a control sample without the CDs, labeled WP-S. For the WP-S-CDs sample, the process involved two main steps. First, a WP solution was prepared by dissolving 5% (*w*/*v*) powder in deionized water. This mixture was heated to 90 °C for 30 min to form a gel, and then its pH was adjusted to 4.8. At the same time, a separate 5% (*w*/*v*) starch (S) solution was made in deionized water, with 1% (*v*/*v*) glycerol added as a plasticizer. This solution was stirred at 60 °C for 30 min. Once both mixtures cooled, the WP gel was combined with the S solution to create a uniform emulsion-forming solution. Finally, garlic-derived CDs were added to this gel at a concentration of 15% (*w*/*w*) based on the WP content. Simultaneously, the WP-S control sample was prepared using the same procedure, but without the addition of CDs. Both the WP-S-CDs and WP-S emulsions were subsequently used for intelligent apple packaging.

### 4.4. Application of Whey Protein–Starch Emulsions Apple Spoilage Sensing

The application of the coating was a four-step process. First, apples were sectioned into four equal pieces to increase the surface area exposed to potential spoilage. These pieces were then fully submerged in the emulsion for 1–2 min to achieve a complete and uniform coating. After being removed, the coated apples were allowed to air-dry at room temperature. The effectiveness of the coating was assessed during room temperature storage by tracking several indicators of freshness. This included monitoring for color degradation and assessing microbial colonies growth over apple.

### 4.5. Characterization

#### 4.5.1. Determination of the pH-Sensitivity of CDs Prepared from Red Garlic Peels

The sensitivity of the garlic-based CDs to pH changes was investigated by monitoring their fluorescence. This was achieved by measuring their fluorescence intensity across a range of pH values. First, buffer solutions were created at pH 2, 7, and 12. The CDs were then introduced into each of these solutions, and the fluorescence emission spectrum was recorded using a spectrophotometer, with a particular excitation wavelength used for all measurements.

#### 4.5.2. Morphological Observation

The prepared emulsions were dried in Teflon plates to form films for investigating by Quanta/250-FEG scanning electron microscope (Thermo Fisher Scientific, Waltham, MA, USA) to obtain SEM images.

#### 4.5.3. Fourier-Transform Infrared (FTIR) Spectra

FTIR spectra were recorded using a Mattson 5000 spectrometer (Unicam, Chard, UK) with KBr pellets.

#### 4.5.4. DFT Calculations

The density functional theory (DFT) calculations were performed using the Gaussian 6.0. 16 09 W software package. The molecular structures were optimized to their lowest energy state using the Berny algorithm. After optimization, several parameters were calculated, including the ground state energies, the energies of the highest occupied molecular orbital (E_HOMO_), the lowest unoccupied molecular orbital (E_LUMO_), and the energy gap (E_g_). Other electronic properties, such as dipole moment (μ), absolute hardness (η), absolute softness (σ), and chemical softness (S), were also determined to understand the CDs’ molecular reactivity and sensing mechanism [4,39].

(1) $$E_{g}=(E_{LUMO}-E_{HOMO})$$

(2) $$\eta =\frac{(E_{LUMO}+E_{HOMO})\;}{2}\;$$

(3) $$\sigma =\frac{1\;}{\eta }$$

(4) $$S=\frac{1\;}{2\eta }$$

## Figures and Tables

**Table 1:** CFU of WP-S and WP-S-CDs.

Microorganism	WP-S	WP-S-CDs
*E. coli*	81.42%	67.44%
*S. aureus*	95.71%	49.42%
*C. albicans*	68.54%	96.63%

**Table 2:** The quantum chemical parameters of WP-S and WP-S-CDs.

DFT	WP-S	WP-S
E_LUMO_ (eV)	0.01180	−0.0172
E_HOMO_ (eV)	−0.1749	−0.1805
E_g_ (eV)	0.1867	0.1633
E_T_ (au)	−2733.35	−3313.06
μ (Debye)	20.04	32.66
ɳ (eV)	−0.0815	−0.098
σ (eV)	−0.1226	−10.11

## Data Availability

Data are available within the manuscript.
